# Trends and predictors of severe and moderate anaemia among children aged 6–59 months in India: an analysis of three rounds of National Family Health Survey (NFHS) data

**DOI:** 10.1186/s12889-024-20328-9

**Published:** 2024-10-14

**Authors:** Vegi Preethi, Vegi Hemalatha, N. Arlappa, MB. Thomas, Abdul Jaleel

**Affiliations:** 1 SRM School of Public Health, Chennai, Tamil Nadu India; 2 ICMR-National Institute of Nutrition (NIN), Hyderabad, Telangana India

**Keywords:** Severe anaemia, Moderate anaemia, Children, NFHS, India

## Abstract

**Background:**

Childhood anaemia remains a significant public health problem in India, as it adversely affects child development and overall health outcomes. This study aimed to analyse the prevalence of severe and moderate anaemia among children aged 6–59 months and identify consistent predictors of this condition over the past 15 years.

**Methods:**

Data from the three most recent rounds of the NFHS were used for this analysis. The final weighted sample included 40,331 children from the NFHS-3 (2005–2006), 200,093 from the NFHS-4 (2015–2016), and 178,909 from the NFHS-5 (2019–2021). Descriptive and bivariate analyses were conducted, followed by binary logistic regression to identify factors associated with severe and moderate anaemia in children aged 6–59 months. All statistical analyses were performed using Stata version 14.

**Results:**

Over the past 15 years, the prevalence of severe and moderate anaemia among children in India has shown a slight decline. However, the influence of various predictors has changed over time. Young children (aged 6–23 months), those from Scheduled Caste (SC) and Scheduled Tribe (ST) communities, and children born to mothers with high parity and low educational attainment remain particularly vulnerable to anaemia. Additionally, short-term illness significantly increases the risk of anaemia. Furthermore, women’s autonomy, indicated by higher education and lower fertility rates, along with maternal nutrition education, have emerged as key factors in reducing anaemia burden in the future. Notably, children whose mothers had no education were 1.4 times more likely to suffer from severe or moderate anaemia. Similarly, children born to mothers with four to five children (OR 1.1, *p* < 0.05) and those with six or more children (OR 1.2, *p* < 0.05) had an elevated risk of anaemia.

**Conclusion:**

The findings highlight three key areas for programmatic focus to accelerate anaemia reduction in India: [1] targeting young children (aged 6–23 months); [2] ensuring the inclusion of SC and ST communities in all relevant interventions; and [3] promoting women’s autonomy. These strategies are essential for reducing the burden of anaemia across the country.

## Introduction

Anaemia, characterised by a low haemoglobin (Hb) concentration and/or a reduced red blood cell (RBC) count, poses significant challenges to an individual’s physiological well-being [1]. Anaemia is caused primarily by imbalances in erythrocyte production and loss, often due to ineffective erythropoiesis influenced by factors such as nutritional deficiencies, inflammation, or genetic haemoglobin disorders [2]. Low Hb levels impair oxygen transport, leading to symptoms such as fatigue, weakness, dizziness, and shortness of breath. While iron deficiency is the leading nutritional cause, deficiencies in folate, vitamin B12, and vitamin A also significantly contribute to this condition [3]. The consequences of anaemia are far-reaching and include increased morbidity and mortality, decreased work productivity [2, 4, 5], and detrimental effects on maternal and child health, including adverse pregnancy outcomes and impaired child development [2, 6, 7].

Anaemia remains a major public health problem in low- and middle-income countries (LMICs), affecting approximately 40% of children aged 6–59 months, 36% of pregnant women, and 30% of women aged 15–49 years [8, 9]. Despite global, regional, and national efforts, progress in reducing anaemia rates has been insufficient to meet the WHO’s target of halving anaemia prevalence by 2030 [10]. In India, anaemia affects 67% of children aged 6–59 months, 59% of adolescent girls aged 15–19 years, and 57% of women aged 15–49 years [11], making it the country with the highest burden of anaemia globally [12–15].

Recognising the severity of this public health problem, the Government of India launched the National Nutritional Anaemia Prophylaxis Programme in 1970, which evolved into the National Iron Plus Initiative (NIPI) in 2011 and later into the Anaemia *Mukt Bharat* (AMB) strategy in 2018. The AMB strategy aims to reduce anaemia across vulnerable populations through its 6 × 6 × 6 approach, which targets six beneficiary groups, implements six interventions, and establishes six institutional mechanisms [16]. Despite these intensive efforts, anaemia remains prevalent, with a notable increase observed from NFHS-4 to NFHS-5 [17]. This highlights the need for a comprehensive understanding of anaemia prevalence, temporal trends, and key predictors to inform targeted interventions in India [18–20].

A PubMed search of recent studies on childhood anaemia using NFHS data yielded three relevant results. The first study examined caste-based inequalities and childhood anaemia using NFHS-3 data, revealing that children from scheduled caste (SC) communities are at a greater risk of anaemia [21]. The second paper conducted a district-level spatial analysis of childhood anaemia utilising NFHS-5 data, highlighting inadequate nutritional supplementation and insufficient healthcare facilities at the district level as significant contributors to childhood anaemia [22]. The third study examined the reversal of the anaemia trend between the NFHS-4 and NFHS-5, identifying maternal education, maternal anaemia status, and socioeconomic status as key drivers influencing changes in anaemia prevalence [17].

In contrast to previous studies that focused on single data points, our study utilised data from the three most recent NFHS rounds to conduct a comprehensive analysis of trends and predictors of childhood anaemia. Additionally, previous studies have focused on overall anaemia prevalence, including mild cases. However, it has been suggested that NFHS may overestimate anaemia prevalence because of its use of the capillary HemoCue method for estimating Hb concentration, which can overestimate anaemia compared with venous blood measurements, especially in mild cases [23]. To address this potential overestimation, our paper specifically analyses severe and moderate anaemia.

The primary objective of this study was to assess the prevalence of severe and moderate anaemia at multiple time points and to examine how it has changed over time. It also sought to identify the population groups at the greatest risk on the basis of their socioeconomic and demographic characteristics. By examining the major predictors of severe and moderate anaemia among children aged 6–59 months, this study aimed to identify consistent predictors across these three survey periods, providing crucial insights for developing effective anaemia reduction strategies in India.

## Data and methods

For this analysis, we used data from three rounds of the nationally representative National Family Health Survey (NFHS): NFHS-3 (2005–06), NFHS-4 (2015–2016), and NFHS-5 (2019–2021). The NFHS is a large-scale, multi-round survey conducted across a representative sample of households in India. The International Institute for Population Sciences (IIPS) in Mumbai, designated by the Ministry of Health and Family Welfare (MOHFW), Government of India, conducts and oversees the survey. NFHS implements a stratified two-stage sampling design across rural and urban areas. Within each stratum, villages/wards were selected on the basis of probability proportional to size (PPS), with explicit stratification on the basis of the percentage of the Scheduled Castes/Scheduled Tribes (SC/ST) population and female literacy rates. The selection of households was determined using a sampling frame compiled from the mapping and listing of households in all primary sampling units (PSUs), covering all states/union territories. The NFHS employs four survey schedules conducted in local languages via computer-assisted personal interviewing (CAPI). To ensure the validity and reliability of estimates related to sociodemographic, health, and biomedical indicators, the NFHS implemented a variety of data quality assurance strategies throughout the survey process. For this analysis, we obtained NFHS data from the Demographic Health Survey (DHS) portal, where all DHS data are publicly accessible upon request.

### Methods of anaemia estimation

In the NFHS, trained health investigators collected blood samples for anaemia testing from eligible respondents. For children aged 6–59 months, informed consent was obtained from a parent or guardian prior to the collection of blood samples for Hb testing. In all survey rounds, a drop of capillary blood was taken from a finger prick (from a heel prick for children aged 6–11 months) to measure Hb levels using the HemoCue Hb 201 + analyser [24]. The HemoCue Hb 201 + analyser is a portable, battery-operated device that self-calibrates before each test and provides immediate onsite results, which are promptly shared with respondents. The consistent use of capillary blood and the HemoCue Hb 201 + analyser across NFHS surveys enables the monitoring of changes in anaemia prevalence over time. Anaemia levels in NFHS were estimated after adjusting for altitude and smoking following protocols developed by the CDC, Atlanta.

### Outcome and explanatory variables

In the NFHS, the prevalence of any anaemia among children aged 6–59 months is defined by a Hb concentration less than 110 g/L adjusted for altitude. This paper focuses specifically on severe and moderate anaemia (Hb concentration less than 100 g/L) for two reasons: [1] Considering the high prevalence of anaemia among children in India, prioritising efforts to address moderate to severe cases is more appropriate, and [2] there are ongoing debates regarding the accuracy of Hb measurement methods employed in the NFHS (capillary HemoCue). Ideally, Hb measurement should be conducted in well-equipped clinical laboratories using venous blood [25–27]. Critics argue that caution is necessary when analysing and interpreting anaemia estimates from NFHS, as the HemoCue device used for Hb measurement may limit the reliability of Hb levels and anaemia prevalence estimates [15]. A recent study conducted in India among children and adolescents aged 1–19 years [28] reported that the average bias for venous HemoCue was 3.0 ± 4.0, whereas the bias for capillary HemoCue was − 3.0 ± 11.0. We assume that if a more reliable method, such as venous blood testing is implemented in the NFHS, the true prevalence of anaemia would match the combined prevalence of severe and moderate anaemia derived from the capillary HemoCue method.

For the final analysis, we included children aged 6–59 months with valid Hb values. We categorised the continuous variable of Hb values (g/L) into two categories. Children with Hb values less than 100 g/L were coded as 1, indicating severe or moderate anaemia, whereas those with Hb values of 100 g/L or higher were coded as 0, indicating mild or no anaemia. This dichotomous variable was considered the outcome variable in this analysis. We included 19 contextually relevant explanatory variables, broadly categorised into child characteristics, household characteristics, nutrition service variables, maternal attributes, and geographic aspects, to investigate their potential influence on predicting severe/moderate anaemia among children across three rounds of the NFHS.

### Sample size, statistical analysis and software used

For this analysis, we applied sampling weights to ensure representative weighted samples and proportions. Sampling weights are adjustment factors that account for differences in the probability of selection, as certain areas or subgroups are often sampled with unequal probabilities. It is recommended to apply weights when tabulating statistics to ensure proper representation. The final weighted sample for this study included 40,331 children in the NFHS-3 (2005–06), 200,093 in the NFHS-4 (2015–16), and 178,909 in the s NFHS-5 (2019–21). We employed descriptive, bivariate, and multivariable analyses. The bivariate analysis allowed us to assess the prevalence of severe and moderate anaemia among children in relation to key demographic, socioeconomic, maternal, and nutritional service variables. To identify factors predicting severe or moderate anaemia among children aged 6–59 months, we used binary logistic regression with 19 covariates. Given the large sample size, logistic regression was well suited for national-level estimates, as it ensures stable parameter estimates and reliable inferences. Binary logistic regression is a widely used statistical method that models the relationship between a binary dependent variable and one or more independent variables. This technique estimates the probability of the dependent variable falling into a specific category (e.g., 1) on the basis of the values of the independent variables, assuming a linear relationship between the independent variables and the log odds of the dependent variable. For this analysis, we used binary logistic regression to calculate the adjusted odds ratio (AOR), controlling for potential confounding factors. All the statistical analyses for this study were conducted using Stata-14, a software package developed by Stata Corp. (College Station, TX, USA).

## Results

### Trends and prevalence of severe and moderate anaemia among children aged 6–59 months

**Fig. 1:**
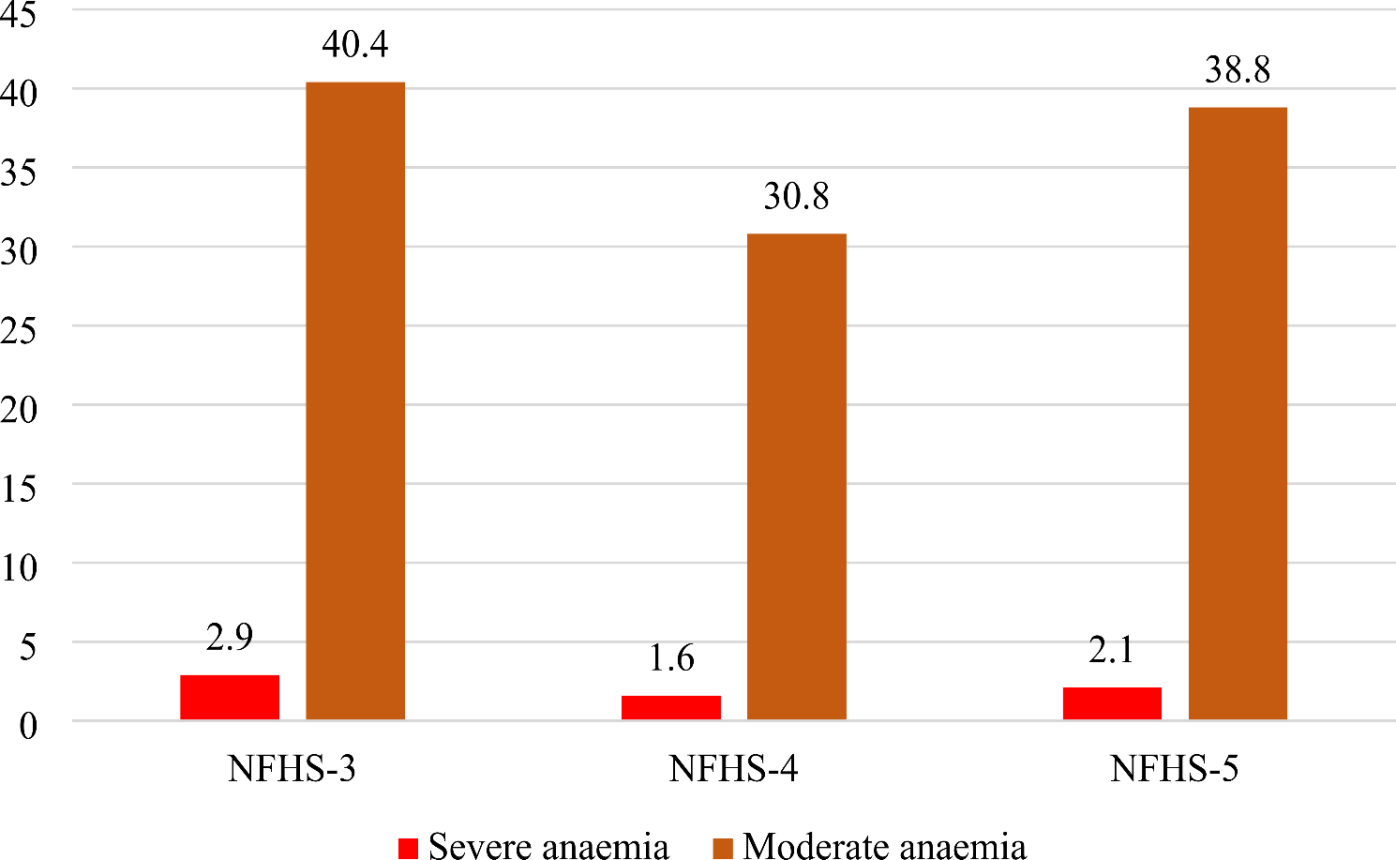
Trends in the prevalence of severe and moderate anaemia (%) among children aged 6–59 months in India

Figure 1 shows the trends and prevalence of severe and moderate anaemia among children aged 6–59 months in India. The prevalence of severe anaemia was 2.9% in NFHS-3, 1.6% in NFHS-4, and 2.1% in NFHS-5. The prevalence of moderate anaemia was 40.4% in NFHS-3, which decreased to 29.2% in NFHS-4, and then increased to 36.7% in NFHS-5. Over a period of 15 years (from the NFHS-3 to the NFHS-5), there was only a slight decrease in the overall prevalence of severe and moderate anaemia among children. However, a notable reversal trend observed between NFHS-4 and NFHS-5, with an increase in anaemia prevalence during this period.

We conducted a bivariate analysis (Table 1) to gain preliminary insights into the clustering of severe and moderate anaemia among children on the basis of demographic, socioeconomic, and maternal factors. The highest prevalence of severe anaemia was observed among young children aged 12–23 months across all three rounds of the NFHS, with rates of 4.5% in the NFHS-3, 2.6% in the NFHS-4, and 3.5% in the NFHS-5. Similarly, the prevalence of moderate anaemia was also higher among children within this age group. Moreover, an increasing trend in both severe and moderate anaemia was observed among very young children (aged 6–11 months) in the NFHS-5 compared with the NFHS-4. In terms of the sex of the child, a slightly higher prevalence was found among males than females across all three survey periods. Furthermore, children who had experienced diarrhoea or fever within the 15 days preceding the survey showed higher rates of both severe and moderate anaemia. Children residing in urban areas had a higher prevalence of severe anaemia than did those in rural areas across the entire survey period. Additionally, children from the SC and ST communities showed a higher prevalence of severe anaemia, with rates of 3.6% and 3.3%, respectively, in the NFHS-3 and 2.5% and 2.2%, respectively, in the NFHS-5. Children born to younger mothers aged 15–19 years consistently showed a higher prevalence of severe anaemia across all three survey rounds, with rates of 4.6% in the NFHS-3, 1.7% in the NFHS-4, and 2.5% in the NFHS-5. Additionally, children of mothers with no education or only primary education consistently had a higher prevalence of severe anaemia across all three survey rounds.

**Table 1:** Prevalence of severe and moderate anaemia among children aged 6–59 months in India by demographic, socioeconomic, maternal, and access to nutrition service characteristics

	NFHS-3 (2005–2006)			NFHS-4 (2015–2016)			NFHS-5 (2019–2021)		
	Severe	Moderate	Total (*N*)	Severe	Moderate	Total (*N*)	Severe	Moderate	Total (*N*)
***N***	**1,178**	**16,299**	**40,331**	**3,120**	**58,532**	**2,00,093**	**3,789**	**65,655**	**1,78,909**
**Total Prevalence**	**2.9**	**40.4**	**43.3**	**1.6**	**29.2**	**30.8**	**2.1**	**36.7**	**38.8**
***Age of the child (in months)***									
6–11	2.0	51.1	2,220	1.8	37.6	8,275	2.6	47.0	9,481
12–23	4.5	54.8	5,297	2.6	40.4	19,302	3.5	49.4	20,669
24–35	3.8	44.2	4,281	1.7	31.9	14,890	2.5	40.0	16,723
36–47	2.7	33.1	3,296	1.1	23.2	11,193	1.5	29.7	12,444
48–59	1.2	25.0	2,383	0.7	17.5	7,991	0.8	24.0	10,128
***Sex of the child***									
Male	3.2	40.5	9,323	1.6	29.6	32,578	2.2	36.8	36,204
Female	2.6	40.3	8,155	1.5	28.9	29,074	2.0	36.6	33,241
***Children who have experienced diarrhea within the past 15 days***									
No	2.8	39.6	15,544	1.5	28.6	54,682	2.0	36.2	63,420
Yes	4.0	48.2	1,930	2.4	35.9	6,970	3.0	42.8	6,024
***Children who have experienced fever within the past 15 days***									
No	2.8	40.0	14,561	1.5	28.7	52,348	2.1	36.2	59,183
Yes	3.3	43.1	2,912	2.2	33.2	9,304	2.3	39.5	10,261
***Type of place of residence***									
Urban	3.1	34.3	3,651	1.7	27.6	16,290	2.2	33.7	16,841
Rural	2.9	42.4	13,827	1.5	30.0	45,362	2.0	37.7	52,603
***Religion***									
Hindu	2.9	41.0	13,956	1.6	29.4	48,846	2.2	37.2	56,227
Muslim	2.8	38.6	2,737	1.6	29.7	10,326	1.8	35.2	10,485
Christian and Others	2.9	38.4	784	1.1	24.6	2,479	2.6	32.4	2,731
***Ethnicity***									
Scheduled Caste (SC)	3.6	44.1	3,990	1.7	30.7	14,085	2.5	39.0	17,277
Scheduled Tribes (ST)	3.3	47.7	1,970	1.5	33.4	7,253	2.2	42.6	8,140
Other Backwards Classes (OBC)	3.1	40.6	7,140	1.6	29.4	27,557	1.9	35.3	28,834
Others	2.2	35.1	3,924	1.5	25.9	10,568	2.2	34.7	11,673
***Wealth Category***									
Poor	3.1	40.4	9,297	1.5	31.7	31,580	2.0	39.4	32,922
Middle	3.4	39.9	3,466	1.8	29.3	12,338	2.3	36.1	13,564
Rich	2.3	34.0	4,714	1.4	25.6	17,735	2.2	33.3	21,439
***Type of source of drinking water***									
Not improved	2.7	41.7	14,939	1.5	29.7	9,074	2.3	36.6	8,961
Improved	2.9	40.1	3,538	1.6	29.2	52,578	2.1	36.6	50,907
***Type of sanitation facility***									
Not improved/no facility	3.1	43.4	12,960	1.6	31.9	34,924	2.2	40.2	22,422
Improved	2.4	33.6	4,492	1.5	26.4	26,728	2.1	35.2	47,023
***Children who received supplementary nutrition from ICDS***									
No	-	-		1.6	30.2	39,053	2.2	37.6	43,693
Yes	-	-		1.3	28.4	11,089	2.1	38.0	16,523
***Children who received IFA syrup***									
No	2.9	40.5	16,713	1.6	29.6	46,316	2.8	45.1	28,543
Yes	2.3	38.8	753	1.4	28.2	15,336	3.0	44.7	18,361
***Mothers’ age (in years)***									
15–19	4.6	52.9	1,277	1.7	33.6	1,520	2.5	47.0	1,754
20–29	2.7	40.5	11,959	1.6	30.0	44,128	2.2	38.0	43,657
30–39	2.9	37.7	3,869	1.5	28.0	14,581	2.0	34.0	13,396
40–49	5.2	34.7	372	1.8	30.0	1,423	1.7	32.1	3,334
***Number of children ever born***									
1	2.6	41.9	3,473	1.4	28.7	15,220	2.2	37.7	18,542
2–3	2.8	38.9	9,035	1.5	28.5	36,864	2.1	36.0	41,518
4–5	3.4	42.3	3,604	1.9	32.6	8,408	2.2	38.0	7,847
6+	3.6	42.2	1,363	2.0	34.0	2,160	2.1	39.0	1,537
***Education of the mother***									
No education	3.5	44.8	9,668	1.9	34.3	22,041	2.4	41.0	16,571
Primary	2.9	39.5	2,451	1.6	30.7	9,157	2.2	39.2	9,212
Secondary	2.2	35.7	4,768	1.4	26.8	25,627	2.0	36.1	34,839
Higher	1.4	28.8	590	1.1	22.7	4,827	1.8	31.0	8,822
***Anaemia status of the mother***									
Severe anaemia	10.6	49.3	422	6.6	46.5	1,007	6.0	48.7	2,185
Moderate anaemia	5.6	50.1	3,953	2.8	41.0	12,292	2.7	43.5	25,455
Mild anaemia	2.7	42.9	7,534	1.5	31.5	27,510	2.0	36.9	18,257
Not anaemic	1.6	33.1	5,568	1.1	22.9	20,634	1.5	30.5	22,882
***Mothers who consumed IFA during pregnancy***									
No	3.5	47.8	4,900	2.0	34.4	11,689	2.4	41.0	6,939
Yes	2.7	40.3	7,949	1.5	29.6	35,318	2.2	38.1	47,284
***Mothers who received nutrition education during pregnancy***									
No	3.2	43.5	1,977	1.6	31.4	10,913	1.8	37.0	7,569
Yes	2.9	39.1	1,709	1.4	28.8	23,730	2.1	37.2	42,999
***Regions***									
North	5.3	38.6	2,393	2.3	32.6	9,208	2.5	35.0	5,404
Central	3.4	44.9	5,498	2.1	33.8	19,552	1.6	32.0	8,034
East	1.6	39.6	4,514	1.0	28.5	15,380	2.6	30.4	484
North east	1.8	35.3	546	0.4	12.3	870	2.5	39.0	14,431
West	2.6	40.1	2,083	1.3	26.3	6,985	1.6	37.7	11,519
South	2.7	36.6	2,444	1.3	26.1	9,525	2.7	35.8	2,202

### Predictors of severe and moderate anaemia among children aged 6–59 months

Table 2 presents the findings from the binary logistic regression analysis. In the NFHS-3, younger children (aged 6–11 months and 12–23 months) had significantly greater odds of experiencing severe or moderate anaemia than did children aged 48–59 months, with adjusted odds ratios of 4.1 and 5.5, respectively. For the NFHS-4, the odds ratio decreased to 2.9 for children aged 6–11 months and 3.3 for those aged 12–23 months. Data from the NFHS-5 indicated that children aged 6–11 months were 1.1 times more likely to experience severe or moderate anaemia, whereas those aged 12–23 months were 0.7 times less likely to experience these conditions.

**Table 2:** Multivariable associations (binary logistic regression) between severe/moderate anaemia among children under five years of age and demographic, socioeconomic and nutritional service variables

	NFHS 3 (2005–2006)		NFHS 4 (2015–2016)		NFHS 5 (2019–2021)	
	Odd Ratio (95% CI)	*p* value	Odd Ratio (95% CI)	*p* value	Odd Ratio (95% CI)	*p* value
***Age of the child (in months)***						
48–59	1					
6–11	4.1 [3.21, 5.11]	< 0.001	2.9 [2.68, 3.08]	< 0.001	1.1 [1.11, 1.22]	< 0.001
12–23	5.5 [4.45, 6.80]	< 0.001	3.3 [3.09, 3.50]	< 0.001	0.7 [0.69, 0.76]	< 0.001
24–35	3.4 [2.78, 4.28]	< 0.001	2.2 [2.05, 2.33]	< 0.001	0.7 [0.38, 1.32]	0.282
36–47	1.9 [1.50, 2.36]	< 0.001	1.3 [1.21, 1.39]	< 0.001	0.6 [0.41, 1.08]	0.107
***Sex of the child***						
Male	1					
Female	0.8 [0.72, 0.90]	< 0.001	0.9 [0.88, 0.94]	< 0.001	0.9 [0.87, 0.94]	< 0.001
***Children who have experienced diarrhoea within the past 15 days***						
No	1					
Yes	1.1 [0.96, 1.37]	0.113	1.05 [0.99, 1.11]	0.072	1.1 [1.09, 1.24]	< 0.001
***Children who have experienced fever within the past 15 days***						
No	1					
Yes	1.1 [0.97, 1.32]	0.092	1.1 [1.01, 1.11]	< 0.05	1.0 [0.95, 1.05]	0.855
***Type of place of residence***						
Urban	1					
Rural	0.9 [0.77, 1.10]	0.416	0.9 [0.89, 0.97]	< 0.05	1.02 [0.97, 1.07]	0.395
***Religion***						
Hindu	1					
Muslim	0.9 [0.71, 1.1]	0.321	1.1 [1.00, 1.12]	< 0.05	0.9 [0.91, 1.02]	0.308
Christian & Others	1.1 [0.88, 1.47]	0.315	0.9 [0.86, 1.00]	0.059	0.8 [0.74, 0.90]	< 0.001
***Ethnicity***						
Others	1					
SC	1.2 [1.00, 1.47]	< 0.05	1.1 [1.01, 1.13]	< 0.05	1.1 [1.00, 1.13]	< 0.05
ST	1.5 [1.24, 1.90]	< 0.001	1.2 [1.14, 1.30]	< 0.001	1.2 [1.08, 1.26]	< 0.001
OBC	1.1 [0.93, 1.33]	0.216	1.1 [1.05, 1.16]	< 0.001	0.9 [0.91, 1.02]	0.255
***Wealth Category***						
Rich	1					
Middle	1.1 [0.87, 1.25]	0.651	1.1 [1.01, 1.12]	< 0.05	1.0 [0.94, 1.05]	0.986
Poor	1.3 [1.05, 1.54]	< 0.05	1.1 [1.06, 1.19]	< 0.001	1.03 [0.97, 1.08]	0.302
***Type of source of drinking water***						
Improved	1					
Not Improved/no facility	0.9 [0.83, 1.08]	0.469	0.9 [0.95, 1.04]	0.960	0.9 [0.85, 0.94]	< 0.001
***Type of sanitation facility***						
Improved	1					
Not Improved	1.2 [1.00, 1.41]	< 0.05	1.03 [0.99, 1.08]	0.119	1.04 [0.99, 1.08]	0.080
***Children who received supplementary nutrition from ICDS***						
Yes	1					
No	-	-	0.9 [0.90, 0.97]	< 0.001	0.9 [0.92, 1.00]	0.082
***Children who received IFA syrup in last six months***						
Yes	1					
No	0.8 [0.62, 0.99]	< 0.05	1.02 [0.99, 1.06]	0.130	1.1 [0.96, 1.03]	0.957
***Mothers’ age (in years)***						
40–49	1					
15–19	1.1 [0.68, 1.82]	0.662	0.9 [0.78, 1.09]	0.374	1.2 [0.96, 1.44]	0.099
20–29	0.8 [0.52, 1.23]	0.313	0.9 [0.81, 1.06]	0.297	1.1 [0.94, 1.33]	0.240
30–39	0.9 [0.58, 1.35]	0.602	0.9 [0.78, 1.02]	0.102	1.0 [0.85, 1.2]	0.962
***Number of children ever born***						
1	1					
2–3	1.2 [1.04, 1.37]	< 0.05	1.1 [1.05, 1.13]	< 0.001	1.0 [1.05, 1.14]	< 0.001
4–5	1.4 [1.10, 1.67]	< 0.05	1.2 [1.08, 1.24]	< 0.001	1.1 [1.01, 1.18]	< 0.05
6+	1.1 [0.80, 1.57]	0.482	1.2 [1.09, 1.41]	< 0.001	1.2 [1.06 1.46]	< 0.05
***Education of the mother***						
Higher	1					
No education	1.2 [0.76, 1.74]	0.502	1.5 [1.35, 1.58]	< 0.001	1.4 [1.33, 1.54]	< 0.001
Primary	1.1 [0.71, 1.66]	0.676	1.3 [1.21, 1.42]	< 0.001	1.3 [1.25, 1.46]	< 0.001
Secondary	1.1 [0.71, 1.60]	0.744	1.1 [1.06, 1.21]	< 0.001	1.1 [1.12, 1.25]	< 0.001
***Anaemia status of the mother***						
Severe	1					
Moderate	0.6 [0.38, 0.99]	< 0.05	0.7 [0.60, 0.84]	< 0.001	0.7 [0.66, 0.87]	< 0.001
Mild	0.4 [0.26, 0.66]	< 0.001	0.4 [0.38, 0.53]	< 0.001	0.5 [0.48, 0.63]	< 0.001
Not anaemic	0.3 [0.16, 0.41]	< 0.001	0.3 [0.24, 0.34]	< 0.001	0.4 [0.37, 0.48]	< 0.001
***Mothers who consumed iron tablets during pregnancy***						
No	1					
Yes	0.9 [0.78, 1.06]	0.244	0.8 [0.84, 0.92]	< 0.001	0.9 [0.91, 1.03]	0.373
***Mothers who received nutrition education during pregnancy***						
No	1					
Yes	0.8 [0.79, 0.99]	< 0.05	0.9 [0.92, 0.99]	< 0.05	1.0 [1.00, 1.11]	< 0.05
***Regions***						
South	1					
North	1.0 [0.81, 1.29]	0.823	1.2 [1.10, 1.25]	< 0.001	1.0 [0.92, 1.16]	0.464
Central	1.0 [0.86, 1.21]	0.787	1.1 [1.05, 1.17]	< 0.001	0.9 [0.78, 0.97]	< 0.05
East	0.6 [0.54, 0.77]	< 0.001	0.7 [0.69, 0.77]	< 0.001	0.8 [0.70, 1.06]	0.223
North east	0.5 [0.33, 0.74]	< 0.001	0.3 [0.28, 0.37]	< 0.001	1.0 [0.90, 1.12]	0.815
West	1.1 [0.92, 1.34]	0.261	0.9 [0.85, 0.97]	< 0.05	1.0 [0.90, 1.13]	0.685

Across all three rounds of NFHS, female children had a notably lower likelihood of experiencing severe/moderate anaemia than male children did. In the NFHS-5, recent episodes of diarrhoea (within 15 days prior to the survey date) were identified as a significant predictor of severe or moderate anaemia among children. Conversely, in the NFHS-4, the incidence of fever within 15 days prior to the survey emerged as a significant predictor of severe or moderate anaemia.

Among different religious groups, children belonging to Christian or other religions showed a significantly lower likelihood of severe or moderate anaemia than those from the Hindu religion did. Children from the SC and ST communities consistently demonstrated a significantly greater likelihood of experiencing severe or moderate anaemia than did children from the other communities across all three rounds of the NFHS survey. In the NFHS-3 and NFHS-4, children from middle- and poor-income households were more susceptible to severe or moderate anaemia than were children from wealthier backgrounds. However, in the NFHS-5, the wealth status distinction was no longer statistically significant in predicting severe or moderate anaemia.

In the NFHS-4 and NFHS-5, children who received supplementary nutrition through the ICDS program were notably more prone to severe or moderate anaemia than those who did not receive such support. However, there was no significant relationship between children’s access to IFA syrup and the incidence of severe or moderate anaemia. The number of children ever born to mothers emerged as a consistent predictor across all three rounds of NFHS. Compared with mothers with one child, those children born to mothers with 4–5 children had a 1.4-fold increased likelihood of experiencing severe or moderate anaemia in the NFHS-3, a 1.2-fold increased likelihood in the NFHS-4 and a 1.1-fold increased likelihood in the NFHS-5. For both the NFHS-4 and the NFHS-5, maternal education is a crucial factor in predicting severe or moderate anaemia among children in India. Children of mothers with no education or primary or secondary education were significantly more likely to experience severe or moderate anaemia than were children of mothers with higher education. Children of mothers with moderate, mild, or no anaemia were notably less prone to severe or moderate anaemia than children of mothers with severe anaemia were. Additionally, children born to mothers who had received nutrition education during pregnancy were significantly less likely to have severe or moderate anaemia according to the NFHS-3, NFHS-4 and NFHS-5.

## Discussion

Anaemia remains a significant public health problem in India, with a particularly pronounced impact on women and children. Despite ongoing efforts and targeted programs, progress in reducing anaemia prevalence has been limited, with a significant proportion of the population still affected. This study investigated the trends and predictors of severe and moderate anaemia among children under five years of age in India, covering the period from 2005 to 2021. By examining changes in anaemia prevalence and identifying persistent predictors, this study provides valuable insights into the evolving landscape of childhood anaemia in India. These findings can inform the development of more effective interventions aimed at addressing this ongoing public health challenge.

The reduction in anaemia prevalence among children in India from the NFHS-3 to the NFHS-5 has been modest. Severe anaemia decreased from 2.9 to 2.1%, and moderate anaemia slightly decreased from 40.4 to 38.8% between 2005 and 2021. However, there was an increase in severe or moderate anaemia from 2015 to 2016 to 2019‒2021. In all three NFHS rounds, the highest prevalence of severe anaemia was observed among children aged 12–23 months. Additionally, there was an increasing trend in severe and moderate anaemia among children aged 6–11 months in the NFHS-5 compared with the NFHS-4. Binary logistic regression analysis confirmed that younger children (aged 6–11 months and 12–23 months) are more susceptible to severe and moderate anaemia than are those aged 48–59 months, underscoring age as a critical determinant.

Several studies have highlighted the increased risk of anaemia in children under 24 months of age, which is attributed to rapid growth, heightened nutrient requirements, and greater susceptibility to infections [29–31]. The results from this analysis also demonstrated that short-term illnesses, such as fever and diarrhea, significantly increase children’s susceptibility to moderate and severe anaemia. These findings are consistent with other studies, which also identified the current and recent incidence of diarrhea and fever as factors associated with anaemia in children under five years of age [32, 33]. Furthermore, several studies highlight that suboptimal infant and young child feeding practices, characterised by insufficient nutrient density and diversity, are key contributors to iron deficiency, which in turn has detrimental effects on children’s cognitive, socioemotional, and psychomotor development [34, 35]. Young children are specifically vulnerable to these suboptimal feeding practices, as they rely entirely on their caregivers for nutrition. This vulnerability may stem from caregivers’ lack of awareness or education about appropriate IYCF practices [34]. Additionally, frontline healthcare workers may not be sufficiently emphasising or providing sustained support for proper IYCF practices [34, 36, 37]. This study also revealed that children born to mothers who had received nutrition education during pregnancy were significantly less likely to have severe or moderate anaemia according to the NFHS-3, NFHS-4 and NFHS-5. Hence, it is crucial to promote nutrition education and advocate optimal infant and young child feeding practices.

Across all three NFHS rounds, children from the SC and ST communities consistently showed a higher likelihood of experiencing severe or moderate anaemia than did children from the other groups, highlighting social disparities in the prevalence of childhood anaemia. Studies have linked SC and ST communities to higher rates of comorbidities [38], mortality [39], childhood stunting and wasting [40, 41], and child mortality [42] and a higher prevalence of childhood anaemia [41, 43]. These findings highlight the need for culturally sensitive strategies that ensure the inclusion of these communities in routine healthcare programs.

The number of children a mother had was a consistent predictor of severe or moderate anaemia among children across all three NFHS rounds. Children born to mothers with 4–5 children had a 1.4 times greater likelihood of developing anaemia in the NFHS-3, a 1.2 times greater likelihood in the NFHS-4, and a 1.1 times greater likelihood in the NFHS-5 than did children of mothers with one child. Children of mothers with no formal education, or only primary or secondary education, had a significantly greater likelihood of anaemia than did those whose mothers had higher education.

This analysis identified several consistent predictors of severe and moderate anaemia among children in India. These factors include a child’s age (6–11 months and 12–23 months), male gender, belonging to SC/ST communities, having mothers with more than three children, and having mothers with low education. These predictors remained significant across all three rounds of the NFHS. Previous research has also identified these factors as significant predictors of childhood anaemia [44]. However, our analysis demonstrated that these factors are consistent over time, and hence should be addressed through programs to accelerate the reduction of anaemia.

Identifying conclusive predictors of anaemia in India using secondary data presents several challenges. First, anaemia is a complex condition influenced by multiple factors, including nutritional deficiencies, infectious diseases, genetic predispositions, socioeconomic status, and cultural norms. This intricate interplay makes it difficult to accurately pinpoint specific predictors. Furthermore, anaemia prevalence estimates from surveys such as the NFHS or similar large-scale studies lack the granularity needed to identify precise predictors. Many of these data sources do not comprehensively capture all potential factors. For example, data pertaining to dietary intake, micronutrient deficiencies, or environmental influences may be limited or inconsistently collected across regions or demographics. To address these limitations, qualitative research should be conducted to explore the social ecology of severe and moderate anaemia among children and women.

## Conclusion

In conclusion, our analysis of three rounds of NFHS data highlights the persistent issue of severe and moderate anaemia among children aged 6–59 months in India. Tackling this public health problem requires addressing the consistent predictors contributing to anaemia in children. Our findings identify three key areas for programmatic focus to increase anaemia reduction efforts: [1] targeting young children (aged 6–23 months) by providing nutrition education to mothers to promote optimal feeding practices and protecting children from short-term morbidities; [2] ensuring the inclusion of SC and ST communities in all relevant programs, such as those related to livelihood, water and sanitation, and nutrition, through a multisectoral approach; and [3] promoting women’s autonomy, as anaemia reduction is linked to maternal education and lower parity.

## Data Availability

The datasets generated and/or analysed during the current study are available in the DHS repository (https://dhsprogram.com/data/dataset_admin/login_main.cfm).
